# 

**Published:** 1917-03

**Authors:** 


					﻿*VW. XXXII
MARCH, 1917
No. 9
TEXAS
Medical Journal
				

